# *NeuroDance*: study protocol for a non-randomized multicenter study of a mixed-style dance intervention for people with Parkinson’s disease

**DOI:** 10.3389/fnhum.2026.1893119

**Published:** 2026-09-17

**Authors:** Kathrin Rehfeld, Ulrike Groß, Anita Hökelmann, Jana Pelclová, Jana Harvanová, Martina Šlachtová, Dagmar Dupalová, Xanthoula Charalampidou, Evangelos Mantsos, Vasiliki Zisi, Uros Marusic, Manca Peskar

**Affiliations:** 1 Faculty for Humanities, Institute of Sport Science, Otto von Guericke University, Magdeburg, Germany; 2 Faculty of Physical Culture, Institute of Active Lifestyle, Palacký University Olomouc, Olomouc, Czechia; 3 Faculty of Physical Culture, Department of Social Sciences in Kinanthropology, Palacký University Olomouc, Olomouc, Czechia; 4 Faculty of Physical Culture, Department of Physiotherapy, Palacký University Olomouc, Olomouc, Czechia; 5 Department of Physical Education and Sport Science, University of Thessaly, Volos, Greece; 6 Science and Research Centre Koper, Institute for Kinesiology Research, Koper, Slovenia; 7 Department of Health Sciences, Alma Mater Europaea University, Maribor, Slovenia; 8 Biological Psychology and Neuroergonomics, Department of Psychology and Ergonomics, Faculty V: Mechanical Engineering and Transport Systems, Technische Universität Berlin, Berlin, Germany; 9 Faculty of Education, University of Ljubljana, Ljubljana, Slovenia

**Keywords:** dance intervention, motor and cognitive function, neuroplasticity, Parkinson’s disease, quality of life, study protocol

## Abstract

Parkinson’s disease is a progressive neurodegenerative disorder associated with substantial motor and non-motor impairments that significantly reduce functional independence and quality of life. While pharmacological treatments remain essential, they often fail to adequately address complex symptoms such as balance deficits, cognitive dysfunction, and reduced wellbeing. Multimodal, non-pharmacological interventions are therefore increasingly recognized as important complementary approaches. This study protocol introduces *NeuroDance,* a novel, structured dance-based intervention designed to target the multidimensional symptom profile of Parkinson’s disease. *NeuroDance* integrates multiple dance styles—including Country dance, Greek folk dance, Tango, and Polka—with improvisational elements. By combining rhythmic auditory cueing, movement variability, social interaction, and internally generated movement, the intervention aims to promote motor learning, enhance neuroplasticity, and improve psychological wellbeing. *NeuroDance* is a multicenter quasi-experimental study conducted across four European countries. It consists of two sequential phases: a feasibility phase and an exploratory effectiveness phase. The feasibility phase uses a single-group pre–post design to evaluate the acceptability, safety, adherence, and practical implementation of the intervention and assessment procedures. Findings from this phase will inform refinements of the intervention protocol and will be reported separately. The exploratory effectiveness phase uses a controlled pre–post design including a *NeuroDance* intervention group and control group. The 18-week intervention is delivered twice weekly in group sessions and is adapted to participants’ individual functional capacities. Primary outcomes include gait, mobility, coordination, balance, cognitive performance, quality of life and subjective wellbeing, while secondary outcomes encompass fear of falling, and disease-specific symptoms. In addition, selected centers assess neurophysiological adaptations using electroencephalography and magnetic resonance imaging to explore underlying neural mechanisms. By integrating diverse movement demands and emphasizing both externally cued and self-initiated motor activity, *NeuroDance* represents a conceptual advance beyond single-style dance interventions. This study protocol provides the methodological framework for evaluating the feasibility and preliminary effectiveness of *NeuroDance* in people with Parkinson’s disease and will inform future controlled trials and implementation strategies.

**Study protocol registration:**

https://drks.de/search/en/trial/DRKS00038241/details.

## 1 Introduction

### 1.1 The need for multimodal rehabilitation in Parkinson’s disease

Parkinson’s disease (PD) affects motor, cognitive, emotional, and psychosocial functioning simultaneously, creating complex rehabilitation needs that extend beyond the scope of pharmacological treatment alone. Although medication and surgical interventions remain essential components of disease management, many symptoms associated with reduced functional independence and quality of life—including balance impairments, gait disturbances, cognitive dysfunction, fear of falling, depression, and social withdrawal—often require comprehensive multimodal rehabilitation approaches. Consequently, there is increasing interest in interventions that simultaneously target multiple domains of functioning and support long-term participation in meaningful activities.

PD is a progressive neurodegenerative disorder characterized by degeneration of dopaminergic neurons in the substantia nigra, resulting in a broad spectrum of motor and non-motor impairments (Balestrino and Schapira, 2020; Bloem et al., 2021; Kalia and Lang, 2015). Cardinal motor symptoms, including bradykinesia, akinesia, rigidity, tremor, postural instability, and gait disturbances, substantially impair functional mobility and independence (Bohnen et al., 2011; Mickel et al., 2016; Moustafa et al., 2016). In addition, individuals with PD frequently experience executive dysfunction, cognitive slowing, anxiety, fatigue, depressive symptoms, and fear of falling, all of which contribute significantly to reduced quality of life (Biundo et al., 2025; Müller, 2013; Soh et al., 2013). Clinical manifestations typically emerge only after substantial neuronal loss has occurred, limiting opportunities for early therapeutic intervention (Kalia and Lang, 2015).

Although pharmacological and surgical therapies remain central to PD management, their effects are often limited with regard to balance impairments, gait dysfunction, cognitive deficits, and fall prevention (Bohnen et al., 2013; Müller et al., 2019; Witt et al., 2017). Furthermore, long-term dopaminergic treatment is frequently accompanied by motor fluctuations and the wearing-off phenomenon, characterized by the re-emergence of motor and non-motor symptoms before the next medication dose. As disease progression advances, these fluctuations increasingly impair daily functioning and reduce independence, highlighting the need for complementary interventions capable of providing more continuous motor and cognitive support. Consequently, exercise-based and other non-pharmacological interventions have gained increasing attention because of their potential to promote neuroplasticity, motor learning, and functional recovery (Abbruzzese et al., 2016; Boas et al., 2024; Mak et al., 2017; Wanner et al., 2022). Importantly, PD is characterized by impaired automatic motor control, which is often compensated for by increased reliance on attentional and cognitive resources. Neurophysiological evidence demonstrates that cognitive-motor dual-tasking leads to distinct changes in brain activity, including increased theta power reflecting primarily the cognitive load, beta desynchronization associated with motor processing, and alpha desynchronization (Marusic et al., 2023; Peskar et al., 2025; Peskar et al., 2023). These findings highlight the importance of interventions that simultaneously target motor and cognitive systems.

### 1.2 Multimodal mechanisms of dance-based interventions in Parkinson’s disease

Among non-pharmacological approaches, dance has emerged as a particularly promising multimodal intervention because it simultaneously engages motor, cognitive, sensory, emotional, and social processes within a single therapeutic framework (Sharp and Hewitt, 2014; Koch et al., 2019; Lihala et al., 2021; Meulenberg et al., 2023; Stanković and Peskar, 2026). Meta-analytic evidence suggests that dance-based interventions are among the most effective complementary therapies for improving both motor and non-motor symptoms in PD (Zhang et al., 2023). From a scientific perspective, dance can be defined as a structured, intentional, and rhythmic sequence of nonverbal body movements unfolding across space and time and shaped by cultural context (Lihala et al., 2021). The multimodal nature of dance arises from the simultaneous integration of sensorimotor, cognitive, emotional, and social components, thereby activating multiple physiological and neural systems.

A central mechanism underlying the therapeutic effects of dance is rhythmic auditory–motor synchronization, also referred to as entrainment, in which music functions as an external cue to facilitate movement timing and coordination (Thaut, 2015; Nombela et al., 2013; Mak et al., 2017). External auditory cues influence motor output through interconnected neural circuits involving the premotor cortex, supplementary motor area, cerebellum, and basal ganglia (Thaut, 2015; Nombela et al., 2013). Because automatic motor control is impaired in PD due to basal ganglia dysfunction, rhythmic auditory cueing can compensate for deficient internal cueing mechanisms by recruiting alternative cortical and cerebellar pathways (Earhart, 2009; Mak et al., 2017). Consequently, music may serve as an external pacemaker that stabilizes motor planning and facilitates movement execution, thereby activating neural regions that are typically underactive in PD (Krotinger and Loui, 2021). These mechanisms have been associated with improvements in gait parameters, including step length, cadence, walking speed, and balance (Bella et al., 2017; Earhart, 2009). In addition, auditory processing possesses high temporal sensitivity, enabling rapid synchronization between rhythm and movement (Nombela et al., 2013). Music also directs attention and supports concentration on movement sequences, which may further facilitate motor performance in PD (Earhart, 2009).

Cognitive resources are simultaneously engaged through the learning of choreographies, anticipation of movement sequences, spatial organization, timing, and conscious awareness of bodily changes (Koch et al., 2019; Rehfeld et al., 2018). Furthermore, dance involves continuous adaptation to complex and variable movement patterns, including coordinated step–arm sequences and multidirectional movement (Rehfeld et al., 2018; Rehfeld et al., 2017). Such variability represents a fundamental principle of motor learning and may facilitate the development of adaptable and generalizable motor strategies, particularly in PD, where impaired automaticity necessitates greater reliance on attentional and executive control processes (Kleim and Jones, 2008; Spampinato and Celnik, 2021; Meulenberg et al., 2023). Dance additionally incorporates action observation, imitation, motor imagery, and improvisational movement, all of which engage higher-order motor planning processes and frontoparietal networks involved in movement preparation and execution (Bek et al., 2022; Jacobs et al., 2009). Improvisational movement, in particular, may support internally generated movement initiation processes associated with the supplementary motor area, which is commonly affected in PD (Jacobs et al., 2009).

Beyond its motor and cognitive effects, dance provides important emotional and psychosocial benefits for people with PD. Music serves as a powerful emotional stimulus that can induce motor entrainment while activating reward-related neural systems, including the putamen, and promoting the release of dopamine and endorphins, which may enhance motivation, mood, and wellbeing (Krotinger and Loui, 2021; Lihala et al., 2021; Salimpoor et al., 2011; Zatorre, 2015). As an artistic and social practice, dance combines movement, music, and shared participation to create meaningful experiences that support cognitive engagement, emotional expression, and a sense of competence (Douka et al., 2019). These effects may be particularly relevant in PD, where apathy, anxiety, reduced self-confidence, and social withdrawal are common non-motor symptoms. Through partner and group activities, dance fosters social connectedness, belonging, and self-efficacy, while contributing to improvements in emotional wellbeing, participation, and quality of life (Bougiesi and Zisi, 2025; Koch et al., 2019; Kshtriya et al., 2015; Lötzke et al., 2015; Douka et al., 2019; Zilidou et al., 2018).

### 1.3 Dance styles and established dance programmes in Parkinson’s disease

Over the past two decades, several structured dance programs have been developed specifically for individuals with PD. Among the most established is *Dance for PD^®^*, which combines various dance styles in an inclusive artistic framework and has demonstrated positive effects on motor function, balance, mobility, cognition, participation, and quality of life (Hackney and Earhart, 2009; Kalyani et al., 2019; Krotinger and Loui, 2021). *Adapted Argentine Tango* programs have shown particular benefits for gait, balance, mobility, and freezing of gait due to their emphasis on rhythmic stepping, weight shifting, directional changes, and partner interaction (Duncan and Earhart, 2012; McKee and Hackney, 2013). Similarly, *NeuroTango^®^* has demonstrated improvements in quality of life, body awareness, and subjective wellbeing, highlighting the importance of emotional and embodied experiences in addition to motor outcomes (Poier et al., 2019; Beerenbrock et al., 2020). Furthermore, *Dance Movement Therapy* emphasizes creativity, emotional expression, and body awareness and has been associated with benefits for psychological wellbeing, self-perception, and social functioning (Lihala et al., 2021).

Collectively, these programs provide strong evidence that dance can improve not only movement-related symptoms but also cognitive functioning, emotional wellbeing, participation, and quality of life. At the same time, the available programs reveal an important gap in the current literature. Many interventions are primarily centered around a single dance style or a relatively standardized dance framework. While such approaches have demonstrated efficacy, they may not fully exploit the diverse therapeutic potential offered by different dance traditions and movement practices. In addition, most studies have focused predominantly on behavioral and clinical outcomes, leaving the underlying neurophysiological mechanisms comparatively underexplored.

This limitation is particularly relevant given that different dance styles appear to engage complementary therapeutic pathways. Argentine tango emphasizes gait adaptation, balance, and partner coordination; folk and social dances promote rhythm, repetition, memory, and social connectedness; ballet-based approaches strengthen posture, body awareness, and movement control; while expressive and improvisational dance foster creativity, adaptability, emotional expression, and self-exploration (Houston and McGill, 2013; Carapellotti et al., 2020). Integrating these elements within a single program may therefore provide broader stimulation of motor, cognitive, emotional, and social processes than any individual dance style alone.

### 1.4 Functional rationale and concept of *NeuroDance*

To address these limitations, the present study introduces *NeuroDance,* a novel mixed-style dance intervention specifically designed for individuals with PD. Importantly, *NeuroDance* is not intended to replace existing evidence-based dance programs. Rather, it builds upon their demonstrated benefits while introducing several novel features. These include the systematic integration of multiple dance styles with distinct motor and cognitive demands, structured improvisational movement tasks, deliberate variation of sensorimotor challenges, and the inclusion of neurophysiological outcome measures such as electroencephalography and functional magnetic resonance imaging. Through this approach, *NeuroDance* seeks to expand current knowledge regarding the mechanisms and feasibility of multimodal dance-based neurorehabilitation for individuals with PD.

The program systematically combines multiple dance styles, including Country dance, Greek folk dance, Tango, and Polka, with improvisational movement elements. This multimodal approach is intended to target complementary sensorimotor, cognitive, and psychosocial processes through variability in movement patterns, rhythm, spatial organization, coordination demands, and social interaction (see Figure 1).

**Figure 1:**
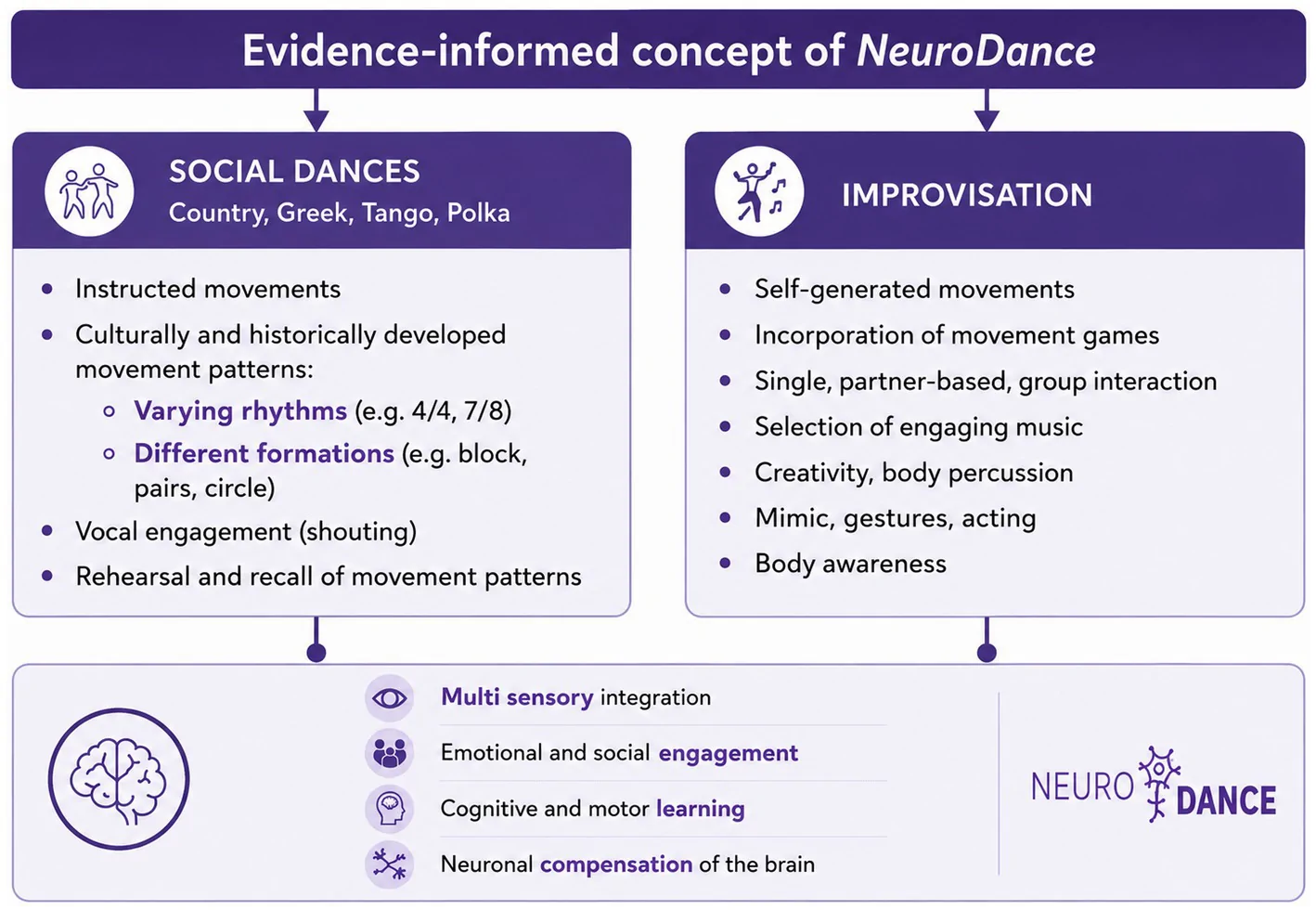
Evidence-informed concept of *NeuroDance*.

The alternating use of different dance styles is designed to promote motor learning by exposing participants to diverse rhythmic structures and movement demands. Country dance may be particularly suitable as an introductory dance form for individuals with PD because it is based on repetitive, rhythmically predictable, and easy-to-follow movement patterns. The clearly structured choreography may facilitate motor learning by providing explicit movement cues, which is particularly relevant in PD, where impaired automaticity increases reliance on attentional control (Löfgren et al., 2019). Repeated step sequences and stable rhythmic patterns may enhance movement confidence, support gait automatization through external auditory cueing, and reduce fear of failure.

In addition, Country dance can easily be adapted to different functional levels by modifying tempo, movement amplitude, or complexity. The side-by-side group format may also lower social anxiety while maintaining important motivational and social components through synchronized group movement. From a neurorehabilitative perspective, Country dance combines rhythmic entrainment, coordinated stepping, directional changes, and balance training within a predictable and accessible framework, making it particularly appropriate for introducing individuals with PD to dance-based interventions (Thaut, 2015; Earhart, 2009; Mak et al., 2017). Although direct clinical studies specifically investigating Country dance in PD remain scarce, this dance form contains several characteristics that are highly relevant for neurorehabilitation.

Traditional Greek dances, characterized by diverse rhythmic sequences and circular group formations, may facilitate gait automatization, rhythmic entrainment, interpersonal coordination, and attentional engagement. Greek traditional dance music is characterized by rhythmic diversity, including asymmetrical meters such as 7/8 and 9/8, which combine shorter and longer rhythmic units into recurring patterns. These distinctive rhythmic structures are closely linked to traditional dance forms and may provide varied temporal cues for movement (Loutzaki, 1994). A previous study has demonstrated that a *Dance for Parkinson’s Disease programme* incorporating Greek dance elements is feasible and may improve quality of life, depressive symptoms, fatigue, cognition, and balance in individuals with PD (Elpidoforou et al., 2022). Long-term participation in traditional Greek dance has additionally been associated with enhanced cognitive functioning, emotional regulation, and social interaction in older adults (Douka et al., 2019), while neurophysiological evidence suggests beneficial effects on cortical network organization and cognitive flexibility (Zilidou et al., 2018).

Argentine tango specifically targets multidirectional stepping, weight shifting, movement initiation and termination, turning, and postural control, all of which directly address motor impairments associated with bradykinesia and postural instability. Tango-based interventions have demonstrated improvements in balance, functional mobility, executive functioning, and spatial cognition in PD (Hackney et al., 2007; McKee and Hackney, 2013).

Polka is traditionally characterized by rapid rhythmic stepping, alternating weight transfer, multidirectional movement, turning, coordinated footwork, dynamic balance demands, and continuous synchronization with music. But the current evidence base for Polka in PD remains indirect and largely theoretical.

A component of *NeuroDance* is improvisation, defined as self-initiated movement without predefined choreography. Improvisational tasks are intended to stimulate internally generated movement processes associated with the supplementary motor area, which is commonly affected in PD (Jacobs et al., 2009). By encouraging autonomous movement generation, improvisation may support impaired internal motor control mechanisms and increase engagement of alternative neural networks. Furthermore, the integration of action observation and motor imagery processes may facilitate movement execution and function as forms of cognitive cueing (Bek et al., 2022).

In addition to motor rehabilitation, *NeuroDance* aims to improve emotional wellbeing, motivation, and social participation through partner-based and group-based activities, positive feedback, and creative movement exploration. Overall, *NeuroDance* represents a multidimensional intervention designed to integrate rhythmic cueing, movement variability, cognitive engagement, improvisation, and social interaction in order to promote neuroplastic adaptation, motor learning, functional performance, and quality of life in individuals with PD.

### 1.5 Objectives and exploratory assumptions

*NeuroDance* is a structured, mixed-style dance intervention for individuals with PD. The study includes a feasibility phase targeting practical implementation, followed by an exploratory effectiveness phase examining preliminary changes in outcome measures after the 18-week intervention. Feasibility results will be reported separately, and effectiveness findings will be interpreted as preliminary and hypothesis-generating.

Specifically, the study aims to:

- Evaluate the feasibility of the *NeuroDance* intervention and the selected assessment procedures, including acceptability, safety, adherence, implementation, and completeness of outcome data.
- Explore whether a multi-style, variability-based dance intervention with integrated improvisation provides a feasible and promising framework for targeting motor, cognitive, psychological, and social dimensions of PD.
- Evaluate motor performance, with a focus on gait, coordination, and dynamic postural control.
- Assess the performance of executive functions, including cognitive flexibility and dual-task performance.
- Examine psychological outcomes, particularly fear of falling and quality of life.

Some centers (SLO, GER) will additionally investigate neurophysiological mechanisms associated with the intervention. These assessments will include electroencephalography (EEG) and functional magnetic resonance imaging (fMRI) to examine changes in functional connectivity, cortical–muscular coupling, and oscillatory markers such as beta–gamma phase–amplitude coupling (Grosse et al., 2002; Zokaei et al., 2021; Braun Janzen et al., 2021; Hodnik et al., 2024; Omejc et al., 2025). Furthermore, cognitive–motor integration and compensatory neural mechanisms will be explored using EEG-based markers of attentional resource allocation and motor processing (Peskar et al., 2025). These neurophysiological assessments are included here for completeness; however, detailed methodology and results will be reported separately.

Given the feasibility and exploratory nature of the study, no formal confirmatory hypotheses are specified. We expect that participants in the *NeuroDance* intervention group may show improvements in motor performance, executive functions, psychological outcomes, and selected neurophysiological markers. These potential changes are expected to be supported by key components of the intervention, including rhythmic cueing, movement variability, improvisation, social interaction, and cognitive-motor engagement. Nevertheless, due to the neurodegenerative nature of the disease posing a general tendency of deterioration of performance, observing no change over time would also be considered a positive finding in terms of maintaining functional independence.

## 2 Methods

### 2.1 Study design

This intervention study consists of a feasibility phase and an effectiveness phase (see Figure 2). The feasibility study (Phase 1) uses a single-arm pre-post design without any control group and aims to examine recruitment, adherence, acceptability, safety, completeness of outcome assessments, and the feasibility of the intervention and assessment procedures. The findings from this phase will be used to refine the intervention protocol and measurement procedures before the controlled phase. Detailed feasibility results will be reported separately.

**Figure 2:**
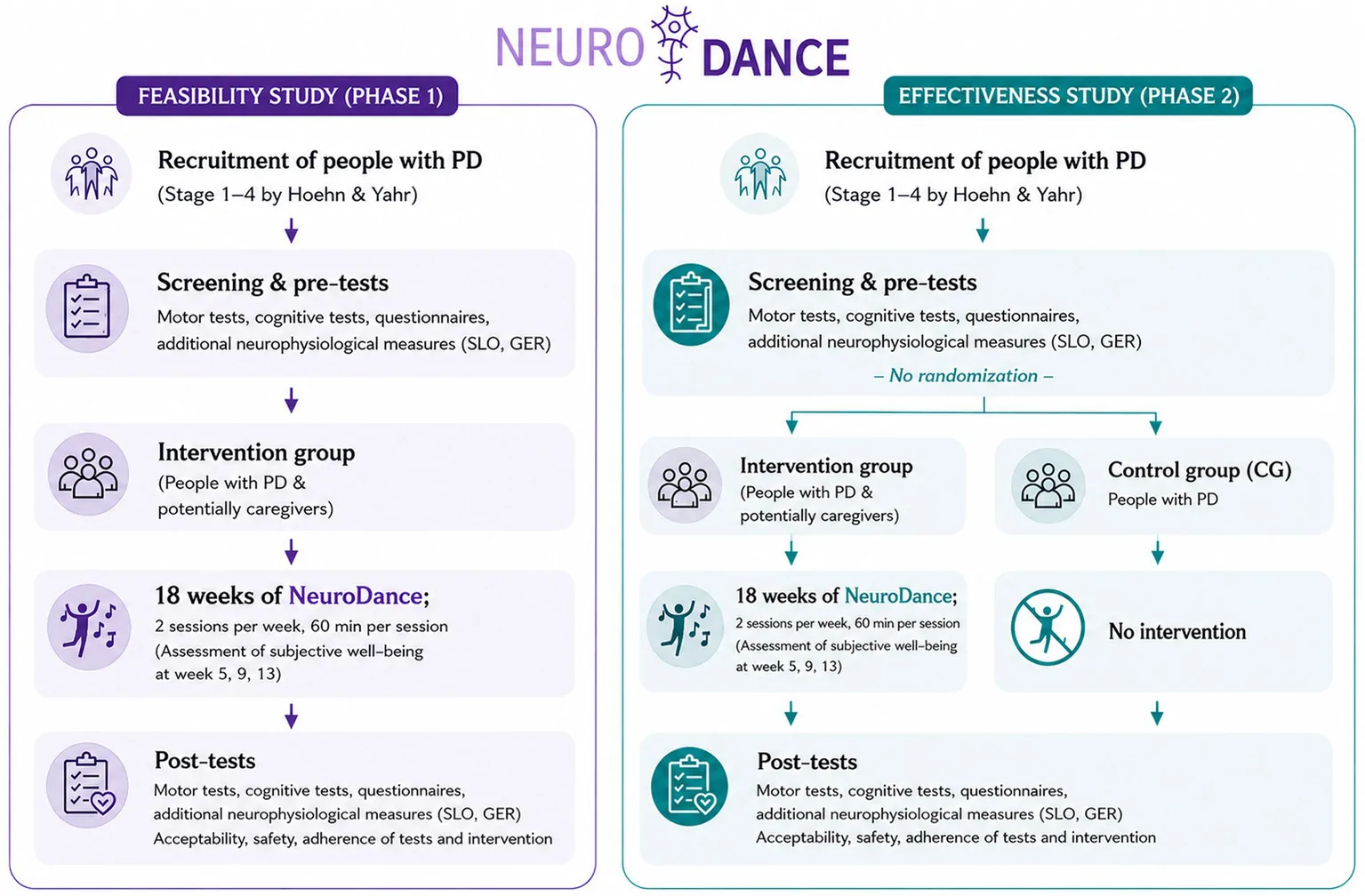
Study design of *NeuroDance* including the feasibility study (Phase 1) and exploratory effectiveness study (Phase 2).

The exploratory effectiveness study (Phase 2) consists of a non-randomized controlled pre-post design and aims to explore preliminary effects of the *NeuroDance* intervention on motor, cognitive, psychological, and neurophysiological outcomes in individuals with PD. Outcomes in the intervention group will be compared with a non-intervention PD control group and, where applicable, with a healthy older adult group undergoing the intervention as the group provides normative context.

The intervention is conducted in four European countries (Slovenia, Germany, the Czech Republic, and Greece) and is therefore designed as a multicenter study. The *NeuroDance* project is funded by the European Union ERASMUS-SPORT-2024 (No. 101185424) and registered in the German Clinical Trials Register (DRKS00038241).

### 2.2 Participants

Participants will be recruited through patient organizations, local newspaper advertisements, social media (Instagram/WhatsApp), and the distribution of flyers in community-based settings, including therapy and care centers.

Participants will be eligible if they meet the following criteria:

- Diagnosis of idiopathic PD confirmed by a neurologist.
- Disease severity corresponding to stages 1–4 on the Hoehn and Yahr scale (Hoehn and Yahr, 1967).
- Stable antiparkinsonian medication (ON-medication phase).
- Age ≥ 40 years.
- Cognitive function allows following the intervention protocol, defined as a score ≥ 21 on the Montreal Cognitive Assessment (MoCA) (Nasreddine et al., 2005).
- Ability to walk independently with or without an assistive device.
- Ability to provide written informed consent.

Participants will be excluded if they present:

- Severe cardiovascular disease or other uncontrolled medical conditions that would preclude safe participation in physical activity.
- Severe autonomic dysfunction or drug-resistant epilepsy associated with an increased risk of falls during physical activity.
- Recent fractures (e.g., hip or femur).
- Chronic severe pain limiting safe participation in movement-based activities.
- Other neurological, musculoskeletal, or psychiatric conditions that would substantially interfere with participation or outcome assessment.
- Inability to meet the defined age or cognitive criteria.

Severe hearing impairment that prevents adequate perception of musical cues or verbal instructions will constitute an exclusion criterion. Speech and language impairments alone will not lead to exclusion provided participants can understand study procedures, follow instructions, and provide informed consent.

Participants with deep brain stimulation (DBS) will be eligible because DBS is increasingly common among individuals with PD and excluding these individuals would limit the ecological validity of the study. DBS status, stimulation parameters, and time since implantation will be documented and considered during interpretation of findings because DBS may independently influence motor symptoms, quality of life, and subjective wellbeing.

### 2.3 The *NeuroDance* intervention

The *NeuroDance* program is a structured, mixed-style dance-based intervention combining Country dance, Greek folk dance, Tango, and Polka, incorporating improvisational elements. The *NeuroDance* intervention will be delivered face-to-face in structured group sessions conducted twice weekly over an 18-week period, with each session lasting 60 min. The 18-week intervention duration was selected based on previous PD-specific dance studies, which typically range from approximately 10–24 weeks, while also allowing sufficient exposure to all planned dance styles and progressive skill development. Furthermore, the duration was established during development of the *NeuroDance* Erasmus+ project framework and was considered feasible across all participating centers while providing adequate time for motor learning, adaptation, and familiarization with the different movement concepts incorporated in the program. A detailed 18-week intervention schedule, including dance styles, selected choreographies, formations, music tempo, and progression, is provided in Table 1. “Jam Sessions” are included between dance-style blocks to rehearse previously learned choreographies, support recall and recognition, and monitor motor learning indicators such as step security, confidence, and ability to perform sequences with reduced instructor guidance.

**Table 1:** Schedule for the 18 weeks of intervention.

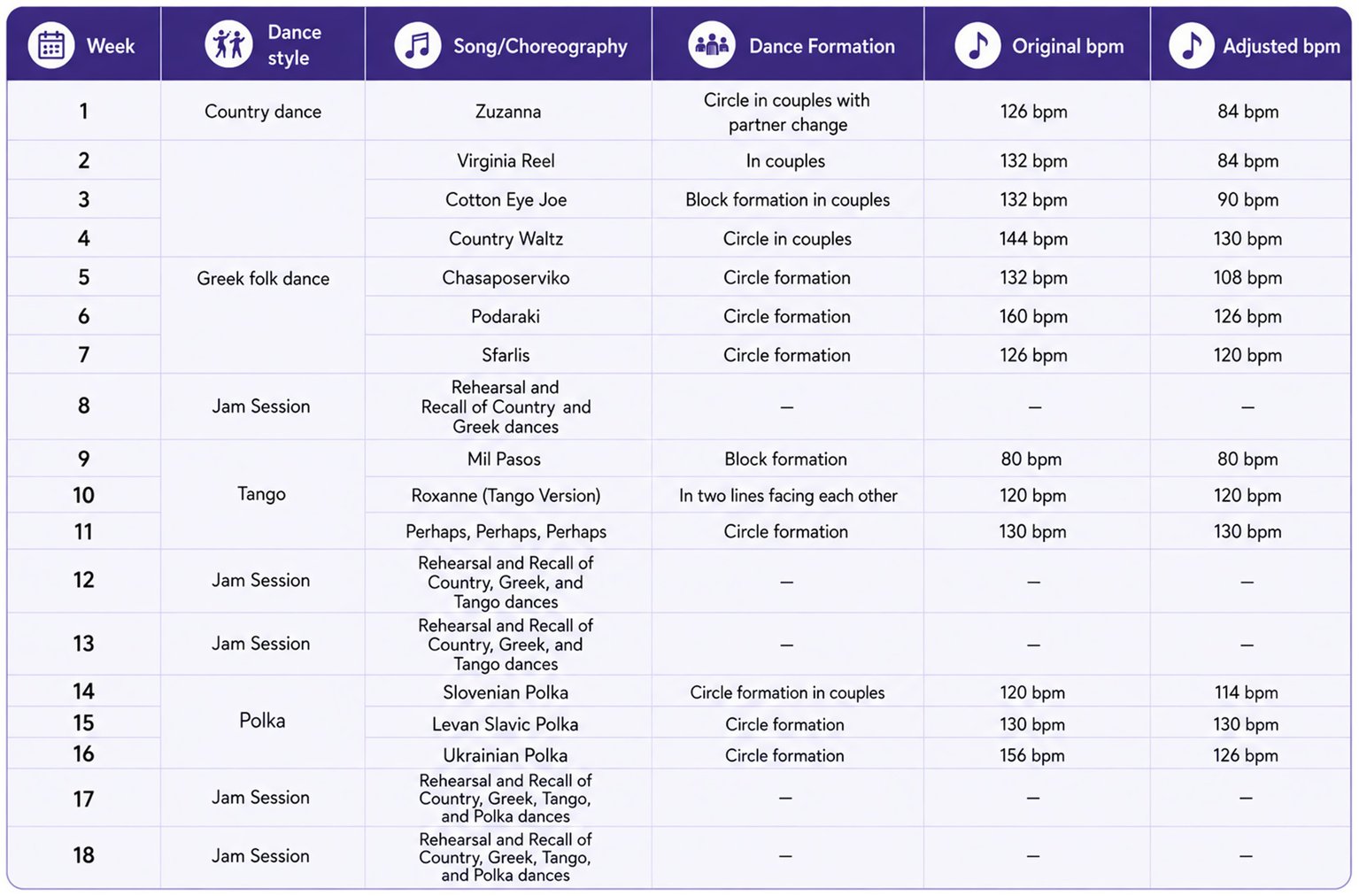

Listed dance styles, songs, dance formation, original tempo in beats per minute (bpm) and recommended adjusted tempo.

Sessions will take place in barrier-free indoor facilities, such as sports halls equipped with mirrors and supportive equipment, with access to adjacent open spaces when appropriate. Each session will follow a standardized structure consisting of four components: a 10-min warm-up, a 10-min improvisation and guided movement exploration phase, 30 min dedicated to teaching and practicing dance sequences, and a 10-min cool-down.

The 10-min warm-up component will be conducted in a seated circle to promote inclusion and group cohesion and will incorporate mobilization, light stretching, rhythmic coordination tasks, breathing exercises, and progressive transitions from seated to standing positions. Typical activities will include seated marching, tapping, clapping, upper-body rotations, stepping patterns, and sit-to-stand movement transition.

The improvisation component will consist of a 10-min segment focused on self-generated movement without predefined choreography, designed to promote internally driven movement initiation and creativity. Participants will be guided using verbal cues, imagery, rhythmic elements, and social interaction, with emphasis on active engagement, adaptability, and individual expression. Movements are intentionally open-ended, allowing participants to modulate amplitude, speed, and complexity according to their abilities, while task instructions provide direction and minimize cognitive load. Improvisation is performed primarily in a standing position with music and includes guided movement exploration based on imagery (e.g., landscapes, astronauts, animals, waves), movement qualities (e.g., light/heavy, slow/fast), and spatial orientation cues. Object-based tasks, such as the use of scarves, facilitate exploration of movement qualities, while simple rule-based tasks (e.g., “stay and go”) introduce variability and require modulation of movement execution. Partner-based improvisation is incorporated through mirroring exercises with alternating leadership and touch-based activities using gentle tactile cues, which influence movement initiation, direction, and quality; all tasks are simplified and paced to ensure safety, accessibility, and sustained engagement.

The choreography teaching and practicing component will last 30 min and consist of two sequential phases. During the first phase, movement sequences will be introduced incrementally to facilitate motor learning and minimize cognitive load. Initial practice will occur without music to emphasize movement accuracy and sequencing, followed by repeated rehearsal with and without music under instructor guidance. Verbal and visual instructional support will be gradually reduced to encourage independence and reliance on auditory cues. Spatial arrangements, including block formations facing a mirror, will be adapted to enhance visual orientation and synchronization, while verbal counting may occasionally be incorporated to reinforce rhythmic processing. In the second phase, participants will practice complete choreographies with progressively reduced external guidance, advancing from instructor-supported execution to increasingly autonomous performance with and without music. Music tempo will initially be reduced where needed and progressively adjusted toward the original tempo over time without explicitly informing participants, thereby minimizing conscious compensation strategies.

The cool-down phase, comprising the last 10 min of the practice session, will be performed in a seated circle with calming music and will include slow controlled movements, gentle stretching, breathing exercises combined with arm movements, and light knee-bouncing exercises to facilitate muscular relaxation. The aim of the cool-down is to gradually reduce physiological and mental arousal, and it can be performed either in standing or seated positions.

All activities will be tailored to accommodate varying levels of motor ability, allowing movements to be performed in seated or standing positions and adjusted in amplitude, speed, and complexity according to individual capacity and daily condition, with external support (e.g., chairs, wall bars) available throughout. Following the feasibility study, substantial modifications to the intervention protocol are planned, while minor real-time adaptations may be implemented as needed to ensure participant safety, comfort, and engagement.

The intervention is delivered by instructors with experience in dance, movement education, or exercise instruction and familiarity with neurological populations. Prior to leading study sessions, all instructors complete a standardized *NeuroDance* certification program developed within the *NeuroDance* consortium. The certification includes theoretical modules covering PD, safety considerations, fall prevention, adaptation strategies, and principles of neurorehabilitation, as well as practical training in all *NeuroDance* components, such as choreography instruction, improvisation techniques, and progression strategies. The program also includes standardization procedures designed to ensure intervention fidelity across participating centers.

Adherence will be monitored throughout the study period using attendance records. Attendance of at least 60% of scheduled sessions will be considered satisfactory adherence and will be used to define the per-protocol sample. Participants with available pre- and post-intervention data will be included in the main exploratory analyses where feasible.

### 2.4 Outcomes

To fulfill the overarching aim of the *NeuroDance* project, a standardized set of motor, cognitive and psychological assessments will be used by all consortium partners. All assessments will be conducted in the practically defined ON-medication state and timed consistently between pre- and post-intervention for each participant. In addition, partners may use site-specific advanced technologies for movement kinematics (e.g., Inertial measurement units—IMUs) and neurophysiological measurements (EEG, fMRI) which will be reported separately. The present study protocol focuses on the primary and secondary outcomes relevant to the project objectives.

#### 2.4.1 Feasibility outcomes

Feasibility outcomes are informed by Bowen and colleagues’ framework for feasibility studies and include recruitment, retention, adherence, acceptability, demand, implementation, practicality, completeness of outcome assessments, occurrence of adverse events, participant and instructor experiences, perceived suitability of intervention components, and implementation-related challenges (Bowen et al., 2009). These outcomes will be analyzed descriptively and reported separately in the feasibility manuscript.

#### 2.4.2 Exploratory effectiveness outcomes

The exploratory effectiveness phase will evaluate preliminary changes in motor, cognitive, psychological, disease-specific, and neurophysiological outcomes following the 18-week *NeuroDance* intervention. The following primary and secondary outcomes are relevant to the exploratory effectiveness phase.

#### 2.4.3 Primary motor outcomes

The primary motor outcomes of the *NeuroDance* project will include gait, mobility, coordination, and balance. The specific task conditions and corresponding outcome parameters are summarized in Table 2.

**Table 2:** Overview of selected motor performance tests, task conditions, and outcome parameters.

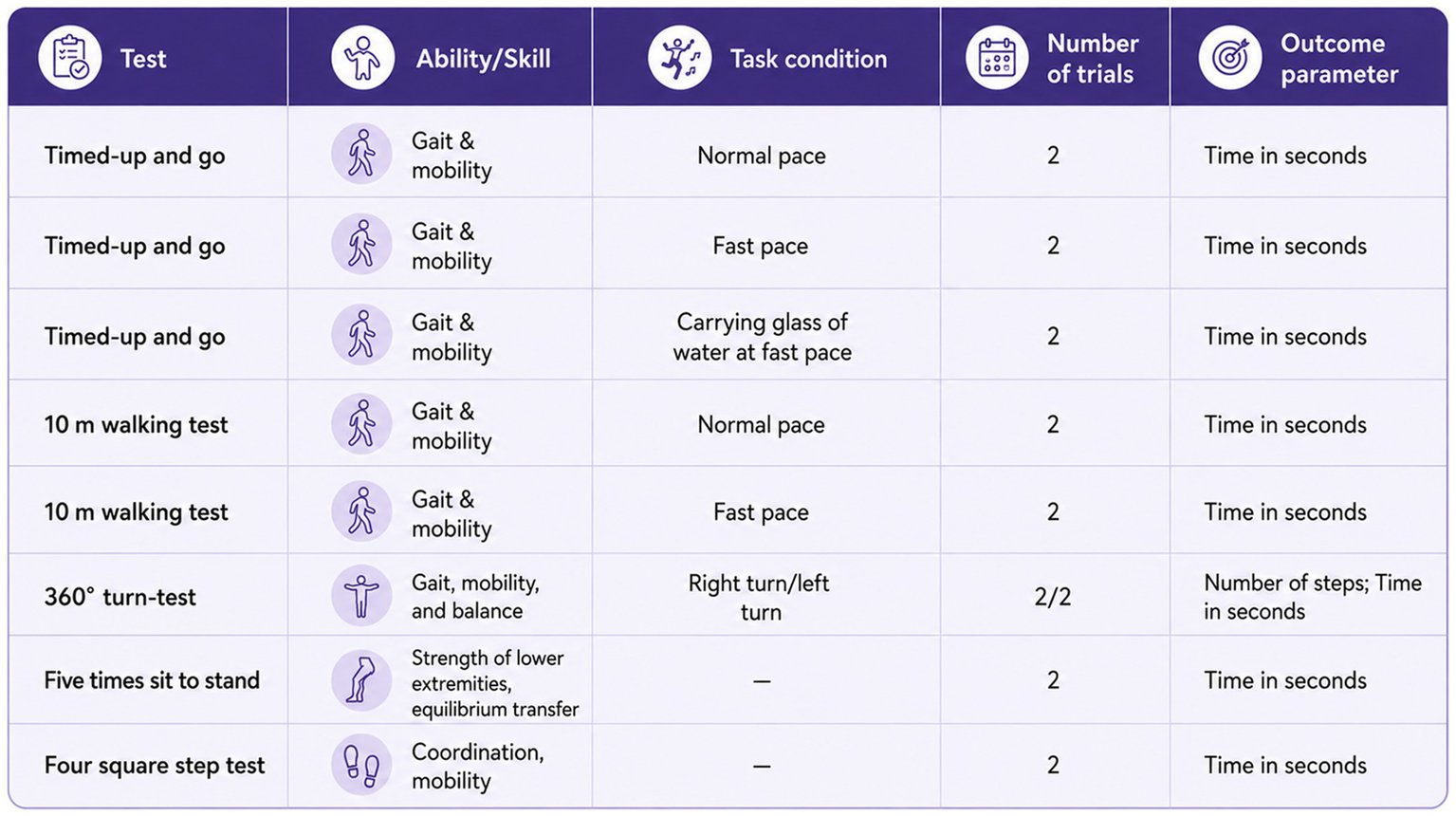

#### 2.4.4 Primary cognitive outcomes

Two cognitive assessments will be included in this project. First, the Montreal Cognitive Assessment (MoCA, see Carson et al., 2018; Nasreddine et al., 2005) is administered as a screening tool to assess global cognitive function. Participants scoring below 21 points are excluded from study participation. Second, the Trail Making Test (TMT: see Reitan, 1958; Tombaugh, 2004) is used to evaluate specific cognitive domains: Part A assesses information processing speed (measured in seconds), whereas Part B evaluates cognitive flexibility (measured in seconds).

#### 2.4.5 Primary psychological outcomes

To assess quality of life and subjective wellbeing, two standardized questionnaires will be employed. The Parkinson’s Disease Questionnaire-39 (PDQ-39) by Peto et al. (1995) evaluates health-related quality of life across eight subdomains: mobility, activities of daily living (ADL), emotional wellbeing, stigma, social support, cognition, communication, and bodily discomfort. A composite score, the PDQ-39 Summary Index (PDQ-39SI), is calculated as the mean of the eight subscale scores, providing an overall measure of quality of life. The PDQ-39 is administered at two distinct time points: pre- and post-intervention.

Subjective wellbeing will be assessed using the WHO-5 Wellbeing Index by Topp et al. (2015), a brief five-item self-report instrument developed by the World Health Organization. It measures subjective wellbeing over the preceding 2 weeks, including aspects such as positive mood, relaxation, vitality, sleep quality, and interest in daily activities. Higher scores indicate better wellbeing, and the instrument also serves as a screening tool for depressive symptoms. In this study, the WHO-5 is obtained at pre- and post-test, as well as during the intervention at weeks 5, 9, and 13.

#### 2.4.6 Secondary outcomes

A range of questionnaires will be selected to comprehensively characterize participants’ demographic background, health status, and disease-specific features, including fall history (FHQ) (Lamb et al., 2005; Lord et al., 2011), fear of falling (FES-I) (Kempen et al., 2008; Kempen et al., 2007; Yardley et al., 2005), freezing of gait (NFOG-Q) (Nieuwboer et al., 2009).

Demographic variables will comprise age, sex, gender, body weight, height, body mass index (BMI), highest educational attainment, occupational status, and relationship status.

Disease-specific data for participants with PD will include age at diagnosis, disease duration, and details of pharmacological treatment.

General health data will include information on the presence of neurological conditions, history of acute head injury, psychiatric diagnoses (e.g., depression, schizophrenia, bipolar disorder), hypertension, diabetes mellitus (type I or II), neoplastic disease, metabolic disorders, cerebrovascular disease, osteoporosis, rheumatological conditions, and osteoarthritis. Information on ongoing non-PD-related medication, physical activity levels, movement behavior, perceived pain, and sensory symptoms is also collected.

Whenever possible, outcome assessments will be conducted by trained assessors who are not involved in intervention delivery. Standardized assessment protocols and validated outcome measures will be used across participating centers. Participants will be informed that responses are confidential and that honest reporting is essential for study quality. These procedures are intended to minimize social desirability bias and assessor-related influences on outcome measurement.

### 2.5 Sample size calculation

The sample size for the feasibility phase was determined pragmatically based on recruitment capacity, expected availability of participants, and the aim of evaluating the acceptability and practical implementation of the intervention and assessment procedures. No formal power calculation was conducted for the feasibility phase, as it is not intended to test intervention effectiveness.

For the exploratory effectiveness phase, an *a priori* power analysis was conducted to estimate the required sample size for detecting a group × time interaction effect on motor performance. Calculations were performed in G*Power 3.1 (Faul et al., 2007) using the “ANOVA: repeated measures, within–between interaction” routine (number of groups = 2, measurements = 2, correlation among repeated measures = 0.5, nonsphericity correction = 1). Assuming a moderate effect size (*f* = 0.25, corresponding to Cohen’s *d* ≈ 0.5), a significance level of *α* = 0.05, and a statistical power of 0.80, a total sample size of *N* = 68 participants (*n* = 34 per group) is required. The power analysis does not include the healthy older-adult group, as this group provides normative context rather than serving as a controlled comparator.

Considering an anticipated dropout rate of approximately 20% due to the 18-week intervention period and the clinical population, the target sample size was increased to N ≈ 85 participants. This conservative estimation ensures sufficient power for detecting clinically meaningful changes in motor performance.

### 2.6 Randomization and blinding

This study is designed as a feasibility and exploratory non-randomized trial and therefore does not employ randomization. A pragmatic allocation approach is used to reflect real-world conditions in individuals with PD, who may experience day-to-day fluctuations in motor and non-motor symptoms. Participants in the intervention group are allocated to available intervention time slots, based on their self-reported morning condition, individual preferences, and logistical feasibility. This approach is intended to support adherence, safety, and ecological validity.

For the exploratory effectiveness phase, allocation is non-randomized. The PD control group comprises participants who continue usual care without the *NeuroDance* intervention during the study period and undergo identical pre- and post-assessments; where ethically and logistically feasible, these participants are offered the intervention after the post-assessment (delayed-intervention/wait-list approach). A healthy older-adult group, where available, provides normative context for the outcome measures rather than serving as a controlled comparator. We acknowledge that a usual-care/wait-list comparator does not control for non-specific effects of group participation, attention, and physical activity. This will be addressed in a future randomized controlled trial with an active comparator.

Allocation concealment is not applicable because group assignment is based on participant preference and study logistics rather than randomization. Blinding of participants and intervention providers is not feasible due to the nature of the behavioral intervention. To minimize assessment bias, outcome assessors will be blinded to group allocation whenever possible. If blinding cannot be maintained, standardized assessment procedures will be applied to ensure consistency. The potential risk of bias associated with the non-randomized design and lack of blinding will be acknowledged when interpreting the findings.

### 2.7 Data management

Data will be collected using standardized assessment forms and subsequently entered into a secure electronic database. Data entry and management will be performed using IBM SPSS Statistics (IBM Corp., Armonk, NY, United States). Statistical analyses will be conducted using the same software. Data quality will be ensured through double-checking of entries, range checks, and plausibility controls.

#### 2.7.1 Data storage

All study data will be stored in a secure, password-protected electronic system accessible only to authorized study personnel. Personal identifying information will be stored separately from research data, and all datasets used for analysis will be pseudonymized. Data will be handled in accordance with applicable data protection regulations, including the General Data Protection Regulation (GDPR). Regular data backups will be performed, and data will be retained for the period required by institutional and legal guidelines.

#### 2.7.2 Handling missing data

The extent and patterns of missing data will be examined and reported. For feasibility outcomes, missing data will be summarized descriptively as part of the evaluation of assessment completeness. For exploratory effectiveness analyses, missing data will be assessed with respect to their likely mechanism. If appropriate, multiple imputation under the missing-at-random assumption will be considered, including relevant outcome variables and covariates in the imputation model. Sensitivity analyses may be conducted to evaluate the robustness of findings under different assumptions regarding missing data.

This approach is consistent with widely accepted guidelines for the analysis of incomplete data in clinical research (Little and Rubin, 2019; Sterne et al., 2009).

### 2.8 Statistical analysis plan

#### 2.8.1 Feasibility study (phase 1)

Feasibility outcomes will be analyzed descriptively. Recruitment, retention, adherence, completeness of outcome assessments, and adverse events will be summarized using frequencies, percentages, means and standard deviations, or medians and interquartile ranges, as appropriate. Participant and instructor acceptability, perceived suitability of intervention components, and implementation-related challenges will be summarized descriptively. Detailed feasibility results will be reported separately.

Exploratory pre–post changes in motor, cognitive, psychological, and disease-specific outcomes during the feasibility phase may be summarized using descriptive statistics and effect sizes with 95% confidence intervals. These analyses will be interpreted as preliminary and hypothesis-generating, and no confirmatory inference will be made.

Where appropriate, exploratory pre–post comparisons may additionally be examined using paired statistical tests (e.g., paired *t*-tests or non-parametric equivalents depending on data distribution). These analyses will be interpreted as exploratory and hypothesis-generating and will primarily serve to estimate potential effect sizes and inform future sample size calculations.

#### 2.8.2 Effectiveness study (phase 2)

Data will be analyzed using linear mixed-effects models (LMMs) to examine the effects of the 18-week dance intervention on motor, cognitive, and psychological outcomes.

For each dependent variable, e.g., gait, mobility, balance, coordination, information processing speed, global cognitive function, cognitive flexibility, quality of life, and subjective wellbeing, a separate model will be specified with:

- Fixed effects for Group (intervention vs. control), Time (pre- vs. post-intervention), and the Group × Time interaction
- A random intercept for participants to account for within-subject correlations across repeated measurements

The primary effect of interest will be the Group × Time interaction, indicating differential change over time between groups.

Pre-intervention values will be inherently accounted for within the repeated-measures structure. If necessary, additional covariates (e.g., age, disease severity) will be included as fixed effects.

## 3 Ethics

The study will be conducted in accordance with the Declaration of Helsinki and applicable national and institutional ethical guidelines, upon approval of the ethical committees of each participating research institute. In Slovenia, the study was approved by the Ethics Committee of the Science and Research Centre Koper (approval number 0624–30/25, approval date 12 March 2025). In Germany, the study was approved by the Ethics Committee of Otto von Guericke University of the Medical Faculty (approval number 115/25, date June 2025). In the Czech Republic, the study was approved by the Ethics Committee of the Faculty of Physical Culture, Palacký University Olomouc (Etická komise FTK UP; approval number: 50/2025; approval date: 23 March 2025). In Greece, the study was approved by the Research Ethics Committee of the University of Thessaly (approval number/date 153/11-11-2025).

All participants will receive written and verbal information about the study aims, procedures, potential benefits, and possible risks. Written informed consent will be obtained before participation. Participants will be informed that participation is voluntary and that they may withdraw from the study at any time without providing a reason and without any negative consequences.

Potential risks related to the dance intervention, such as fatigue, muscle discomfort, dizziness, loss of balance, or falls, will be minimized through individual tailoring, progressive instruction, availability of support equipment, and supervision by trained instructors. Adverse events will be documented and managed according to local procedures.

Personal identifying information will be stored separately from research data, and all datasets used for analysis will be pseudonymized. Data will be handled in accordance with applicable data protection regulations, including the General Data Protection Regulation (GDPR).

## 4 Dissemination

Findings from the *NeuroDance* project will be disseminated through peer-reviewed publications, conference presentations, project reports, professional workshops, and communication activities targeting healthcare professionals, researchers, dance instructors, patient organizations, people with PD, caregivers, and policy stakeholders.

The feasibility findings, including acceptability, adherence, safety, implementation-related challenges, and participant feedback, will be reported separately. Results from the exploratory effectiveness phase will be disseminated as preliminary and hypothesis-generating evidence to inform future randomized controlled trials and implementation strategies.

Public-facing dissemination activities will include project website updates, newsletters, social media communication, patient-oriented materials, demonstration events, and training resources. All dissemination activities will acknowledge EU funding and follow applicable ethical and data protection requirements.

To support sustainability, training materials and implementation resources developed within the project will remain available beyond the project duration wherever possible, and partnerships with healthcare, academic, patient, and cultural organizations will be maintained to facilitate future implementation.

## 5 Discussion

### 5.1 Clinical relevance

The *NeuroDance* program is expected to provide a meaningful and innovative addition to existing rehabilitation strategies for individuals with PD. By targeting core motor impairments such as gait disturbances, reduced functional mobility, and postural instability, the intervention directly addresses key factors associated with loss of independence and increased fall risk.

Importantly, *NeuroDance* may also help mitigate the functional consequences of motor fluctuations, including the wearing-off effect associated with long-term dopaminergic treatment. As symptom severity can vary throughout the day, interventions that promote more stable motor control and compensatory strategies are of particular clinical relevance. In this context, the combination of externally cued movement (e.g., rhythm and music) and internally generated motor activity (e.g., improvisation) may support more consistent functional performance across fluctuating states.

In contrast to conventional exercise-based therapies, *NeuroDance* offers a highly engaging, socially interactive, and intrinsically motivating format, which may enhance adherence and long-term participation. This is particularly relevant given reduced motivation and physical activity levels frequently observed in PD. Furthermore, the integration of emotional and social components may contribute to improvements in wellbeing, self-efficacy, and quality of life, which are often insufficiently addressed in standard care.

If proven feasible and effective, *NeuroDance* could be implemented in both clinical rehabilitation settings and community-based programs, representing a scalable and cost-effective complementary intervention.

### 5.2 Neuroscience implications

From a neuroscience perspective, *NeuroDance* contributes to an emerging framework of multimodal, neuroplasticity-driven interventions in neurodegenerative diseases. The program integrates several mechanisms known to facilitate neuroplastic adaptation, including rhythmic auditory cueing, movement variability, cognitive engagement, and emotional activation (Kleim and Jones, 2008; Krotinger and Loui, 2021; Meulenberg et al., 2023; Thaut, 2015).

Consistent with current evidence, dance-based interventions may promote activity-dependent neuroplasticity, reflected in changes in functional connectivity between motor and cognitive brain networks, including the basal ganglia, cerebellum, and prefrontal cortex (Meulenberg et al., 2023; Rehfeld et al., 2017; Rehfeld et al., 2018). These adaptations are particularly relevant in PD, where degeneration of dopaminergic pathways disrupts internal motor control and necessitates compensatory recruitment of alternative neural circuits (Kalia and Lang, 2015; Roytman et al., 2025).

A key aspect of *NeuroDance* is its potential to enhance cognitive-motor integration. Individuals with PD often rely on increased attentional control to compensate for impaired automaticity of movement (Nombela et al., 2013; Roytman et al., 2025). Interventions that simultaneously engage motor and cognitive processes may therefore optimize the allocation of neural resources and improve task performance. Neurophysiological findings, including changes in theta, beta, and alpha oscillatory activity, suggest that such training may lead to more efficient neural processing and reduced compensatory overload (Peskar et al., 2023; Peskar et al., 2025).

Furthermore, the integration of motor simulation processes—including action observation, imitation, and motor imagery—represents a novel and theoretically grounded component of the *NeuroDance* concept. These processes activate frontoparietal networks involved in motor planning and execution and may facilitate movement initiation and coordination (Bek et al., 2022; Jacobs et al., 2009). Embedding these mechanisms within a creative and ecologically valid context may enhance both transfer to activities of daily living and long-term engagement.

Another important contribution lies in the systematic incorporation of movement variability and improvisation. From a motor learning perspective, variable practice promotes the development of flexible and generalisable motor skills, which are particularly relevant in PD, where rigid and context-specific movement patterns are common (Kleim and Jones, 2008; Spampinato and Celnik, 2021). By continuously challenging the motor system through diverse movement demands, *NeuroDance* may foster adaptability and resilience in motor behavior.

### 5.3 Conceptual advancement

Beyond its clinical and neurophysiological implications, *NeuroDance* may represent a conceptual advancement over existing dance-based interventions. While most previous studies have focused on single dance styles, the present approach systematically combines multiple styles with improvisational elements, thereby increasing the diversity of sensorimotor, cognitive, and emotional demands.

This multimodal design aligns with contemporary theories of enriched and complex environments as drivers of neuroplasticity. The integration of structured movement patterns, creative exploration, and social interaction creates a rich stimulus environment that may maximize therapeutic effects.

In addition, the inclusion of neurophysiological measurements (e.g., EEG, fMRI) serves as an exploratory investigation of potential mechanisms underlying behavioral changes. This addresses an important gap in previous dance-intervention research, which has often focused on behavioral outcomes alone.
